# Hedgehog Promotes Production of Inhibitory Interneurons in Vivo and in Vitro from Pluripotent Stem Cells

**DOI:** 10.3390/jdb4030026

**Published:** 2016-08-26

**Authors:** Nickesha C. Anderson, Christopher Y. Chen, Laura Grabel

**Affiliations:** Department of Biology, Wesleyan University, 52 Lawn Avenue, Middletown, CT 06459, USA; cychen@wesleyan.edu (C.Y.C.); lgrabel@wesleyan.edu (L.G.)

**Keywords:** Sonic hedgehog, GABAergic interneurons, medial ganglionic eminence, pluripotent stem cells

## Abstract

Loss or damage of cortical inhibitory interneurons characterizes a number of neurological disorders. There is therefore a great deal of interest in learning how to generate these neurons from a pluripotent stem cell source so they can be used for cell replacement therapies or for in vitro drug testing. To design a directed differentiation protocol, a number of groups have used the information gained in the last 15 years detailing the conditions that promote interneuron progenitor differentiation in the ventral telencephalon during embryogenesis. The use of Hedgehog peptides and agonists is featured prominently in these approaches. We review here the data documenting a role for Hedgehog in specifying interneurons in both the embryonic brain during development and in vitro during the directed differentiation of pluripotent stem cells.

## 1. Introduction

Glutamatergic projection neurons and gamma-aminobutyric acid-containing (GABAergic) inhibitory interneurons are the two major classes of neurons in the cerebral cortex. Despite constituting only around 20%–30% of the total neuron population in the mammalian cortex, inhibitory interneurons play a key role in modulating the overall activity of this region [1]. Reflecting their various functions, the cortical interneuron population is extraordinarily diverse and can be characterized by multiple measures. These include the expression profile of calcium binding proteins parvalbumin (PV), calbindin (CB), calretinin (CR), and neuropeptides somatostatin (SST), neuropeptide-Y (NPY), vasoactive intestinal polypeptide (VIP), their morphology, site of synapse formation, and electrophysiological properties [2,3,4].

An impaired balance of excitatory and inhibitory activity, often due to the abnormal development, loss of, or damage to the cortical interneuron population, characterizes the pathology of a broad array of neurological disorders, including epilepsy, schizophrenia, autism spectrum disorders (ASD), and Alzheimers Disease (AD) [5,6,7,8]. These observations have stimulated great interest in learning when, where, and how GABAergic inhibitory interneurons are generated in the mammalian brain, and in using this information to establish protocols for generating inhibitory interneurons from pluripotent stem cells (PSCs). In the rodent, it is clear that cortical interneurons derive from transient embryonic structures in the ventral telencephalon, the ganglionic eminences, and then migrate tangentially into the cortex [2,4,9,10]. A variety of experimental approaches, including mutational analysis and lineage tracing, have revealed that the medial ganglionic eminence (MGE) is the primary source of SST and PV-expressing interneurons, whereas, with some exceptions, CR-positive cells arise predominantly in the caudal ganglionic eminence (CGE) [11,12,13]. As described below, Sonic hedgehog (Shh) plays a key role in patterning the ventral telencephalon and establishing the distinct interneuron subtypes [14].

Based upon what has been learned about the extrinsic and intrinsic cues that establish the interneuron lineages in the rodent brain [5,15], protocols have been designed to generate GABAergic inhibitory neurons from PSCs [16,17,18,19,20,21,22]. The ability to generate inhibitory interneurons from a PSC population opens up a number of promising avenues of research. Interneuron-related disease-specific induced pluripotent stem (iPS) cells, isolated from patients or generated by gene editing strategies, could be evaluated for their ability to differentiate into interneurons in culture [23]. These studies may reveal specific deficiencies in interneuron survival, differentiation, or function that could in turn become targets for drug design and help elucidate the cellular and molecular basis of the defect. iPS cells generated from severe idiopathic ASD patients have recently been used to generate cerebral organoids, three-dimensional aggregates that mimic germinal centers and allocation of cell types to specific layers observed in the developing cortex [24]. These ASD patient-specific organoids are characterized by the over-production, relative to control cell lines, of GABAergic inhibitory interneurons [25]. An excess of inhibitory interneurons has also been observed in ASD patient postmortem tissue [26], supporting a role for an imbalance in the ratio of GABAergic/glutamatergic neurons in ASD.

PSC-derived interneuron progenitors can also be used for cell transplantation therapies designed to treat interneuron-associated disorders. Using rodent disease models, transplants containing embryo-derived interneuron progenitors have led the way, and some success, characterized by transplant integration and reversal of disease-specific deficits, has been reported for epilepsy, schizophrenia, AD, Parkinson’s, and ASD (reviewed in [8]) [6,7,8,27]. It is unlikely, however, that sufficient human fetal material would be available for clinical application, leading many groups to turn to human PSC-derived interneuron progenitors as graft material, and disease-specific immune-compromised mice as animal model hosts. One notable success documenting treatment efficacy has been for temporal lobe epilepsy, in which loss of hippocampal interneurons is associated with seizure activity and memory deficits [27]. A number of groups have noted maturation of PSC-derived neural progenitors into interneuron subtypes following transplant to the hippocampus, as well as acquisition of interneuron-specific firing patterns, and integration into the host circuitry [20,21,28,29,30]. Most notably, a recent report has documented the ability of human embryonic stem cell (ESC)-derived interneuron progenitors transplanted to the hippocampus of temporal lobe epilepsy model mice to suppress recurring seizures and improve behavioral deficits [28].

It has long been proposed that the increased cognitive ability of human and non-human primates is associated with an increased complexity of the cortical interneuron population [31,32]. A number of recent observations suggest we cannot simply extrapolate from what has been learned about the origin and diversity of cortical interneurons in rodents to the primate brain. The human fetal ventral telencephalon contains the analogous ganglionic eminence structures observed in the rodent embryonic brain, and they appear to be the major source of cortical interneurons [32,33]. However, the details of interneuron production differ from what is observed in the rodent in a number of aspects [34,35], and a major controversy has arisen over the existence of an additional primate-specific interneuron germinal center located dorsally, in the cortex itself, that generates a subset of cortical interneurons [36]. Such a source could provide the increased abundance and heterogeneity of interneurons observed in the primate brain. Direct comparison of the output of interneurons produced using directed differentiation protocols of mouse versus human PSCs, including use of cerebral organoid protocols that mimic cortical development [24], may be useful for elucidating the human-specific attributes of interneuron production.

Hedgehog signaling plays an essential role in neurogenesis. Vertebrate Hedgehogs act by suppressing the inhibitory effect of the Patched 1 (Ptch1) membrane protein on the effector Smoothened (Smo), resulting in the positive action of Gli transcription factors. In the neurogenic niches of the embryonic and adult central nervous system (CNS), Shh acts via primary cilia to promote the survival and proliferation of neural stem cells [37,38,39,40,41]. Gradients of Shh pattern the developing neural tube, promoting ventral cell type identity in the spinal cord and brain [42,43]. In the developing telencephalon, Shh is expressed at high levels ventrally, and as described below, plays a key role in modulating the production of distinct interneuron subtypes in the embryo and can be used to direct interneuron differentiation of PSCs.

## 2. The Importance of Shh in Specification of Cortical Interneurons in Vivo

The wide variety of GABAergic cortical interneurons in both rodent and primate models originate predominantly in the MGE, CGE, and lateral ganglionic eminences (LGE) [34,35,44,45]. The MGE is the major source of inhibitory interneuron progenitors, generating approximately 60%–70% of all GABAergic cortical interneurons, with the CGE and preoptic area (POA) contributing 30% and 5%–10%, respectively, during rodent corticogenesis [4,45,46,47]. The MGE begins to develop in mice at E10.5 as a neuroepithelial bulge from the walls of the ventral telencephalon into the lateral ventricle, with NKX2.1 strongly expressed throughout this developing structure [12]. By E12.5, GABAergic interneuron progenitors begin their tangential migration from the ganglionic eminences into the cerebral cortex to undergo differentiation and maturation [4]. Expression of LHX6, a transcription factor regulating neuronal migration and maturation, is extended from the MGE at E12.5 to both the striatum and cortex by E14.5 [12]. Recent studies indicate species-specific differences between rodent and primate models in the organization and developmental origins of cortical interneurons. Analysis of fetal tissues collected at different gestational ages from lower primates and humans suggest a much larger contribution of cortical interneurons generated from the CGE and dorsal LGE in primates compared to rodents [34]. Nonetheless, expression patterns and transcription factors regulating the development of the ventral forebrain are similar between primates and rodents, indicating that molecular mechanisms involved in the genesis and maturation of cortical interneurons within the mammalian brain are highly conserved [34,35].

Shh signaling is a requirement for the normal development and patterning of the telencephalon and is one of the best-studied examples of a morphogen, regulating both the spatial arrangement and control of cellular differentiation in numerous developing tissues. During early development of the embryonic telencephalon, Shh establishes a reciprocal gradient with its transcriptional repressor Gli3, bone morphogenetic protein (BMP) and WNT, which are all expressed at higher levels in the dorsal telencephalon [48,49]. The expression of the homeobox transcription factor *NKX2.1* is required for the specification of the MGE, and is maintained by Shh throughout neurogenesis [12]. The induction and maintenance of *NKX2.1* expression by Shh in the MGE is critical for the specification of PV- and SST-expressing interneurons [50,51]. As Shh functions upstream of *NKX2.1*, conditional knockout of Shh in the telencephalon of postnatal day 12 mice results in a significant reduction in the number of progenitors expressing *NKX2.1* in the ventricular zone (VZ) of the MGE, and therefore a loss of PV and SST interneurons observed in the postnatal cortex [38,52]. The loss of *NKX2.1* expression in the ventral telencephalon resulting from the downregulation of Shh signaling initiates dramatic morphological and molecular changes to the MGE. During initial patterning of the MGE, in addition to maintaining *NKX2.1* expression, Shh antagonizes GSX2, a transcription factor that promotes the specification of the vertically oriented, bipolar CR-expressing interneurons derived mainly from the CGE [13,53]. The inactivation of Shh signaling in the developing forebrain results in the concomitant upregulation of GSX2, virtually eliminating S-phase neural progenitor cells that express *NKX2.1* in the ventral telencephalon [14]. The constitutive loss of *NKX2.1* results in a ventral-to-dorsal re-specification, as the MGE acquires markers characteristic of LGE and CGE derivatives [12,14]. In contrast, RT-PCR analysis of human radial glial cells (RGCs) dissociated from the dorsal telencephalon and supplemented with exogenous Shh in vitro shows upregulated expression of downstream Shh target genes, *Gli1* and *Ptc1* [54]. This conversion of PV- and SST-expressing interneurons to CR and VIP-interneuron subtypes that predominately originate from the CGE, and the reprogramming of dorsal to ventral telencephalic identity after Shh exposure, illustrate the plasticity of interneuron fate specification and the role played by Shh within the ventral subpallium.

**Figure 1:**
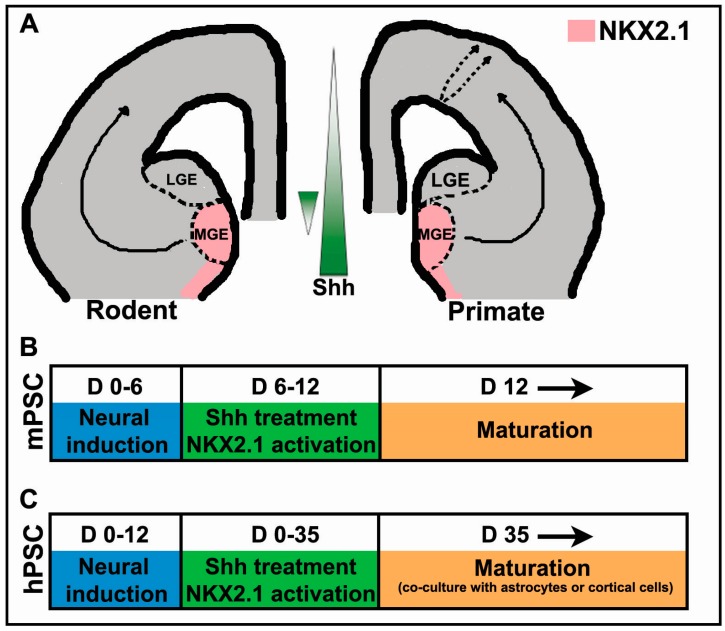
Sonic hedgehog (Shh)-dependent in vivo and in vitro cortical interneuron progenitor specification and maturation. (**A**) Schematic illustration of interneuron fate specification in vivo in the rodent and primate model. Shh signaling is highest in the developing ventral forebrain in contrast to a reversal in this gradient within the medial ganglionic eminence (MGE). Black solid arrows represent established tangential migrations of inhibitory progenitors derived from the MGE and dashed arrows indicate a proposed dorsal niche generating a subset of GABAergic progenitors that move radially to the neocortex. The shaded pink area represents *NKX2.1* expression within the ventral telencephalon; (**B**,**C**) mouse (mPSC) and human pluripotent stem cells (hPSC) differentiation timelines (in days (D)) for deriving GABAergic interneurons in vitro.

While the gradient of Shh, highest ventrally, is essential in establishing the dorso-ventral axis within the rodent forebrain, there is evidence suggesting this gradient is reversed in the MGE in mouse embryos at embryonic day 13.5 (Figure 1A; [55]).

RNA profiling using GeneChip arrays determined that expression of a number of genes implicated in the Shh signaling pathway, including *Gli1/2*, *Ptch-1*, and *Hedgehog-interacting protein 1* (*Hhip1*), is enriched in the dorsal relative to the ventral MGE [55]. Transplantation of S-phase progenitors dissected from the dorsal, as compared with the ventral MGE, yielded an almost 4:1 bias in the generation of SST-positive interneurons. Conversely, transplanted progenitors from the ventral MGE were more than twice as likely to develop into PV-positive interneurons [44,55]. These results strongly suggest that a gradient of Shh signaling generates distinct domains with defined differences in gene expression within the MGE that biases the production of SST or PV-expressing interneurons.

The POA, situated in the rostral forebrain, is an additional source of cortical GABAergic interneurons, producing different classes of interneurons that populate the cerebral cortex in mice. Similar to the MGE, virtually all POA progenitors express *Shh* and *NKX2.1* [44,56]. Lineage tracing experiments in mice further classified progenitors originating from specific subdomains within the POA, expressing either *NKX5.1* or *DBX1*, that undergo a similar tangential migration to the neocortex [46]. However, further experiments are needed to determine whether comparable populations of POA-derived GABAergic interneuron progenitors exist in lower primates and humans.

Apart from the *NKX2.1*-expressing neurogenic niches of the MGE and POA, the CGE, and to a lesser extent the LGE, generate the remaining fraction of cortical interneurons. The CGE, a subcortical domain of the ventral telencephalon that does not express *NKX2.1*, generates a number of distinct interneuron subtypes that preferentially populate the superficial layers of the cortex and express the type 3 serotonin receptor 5-HT3AR [9,57]. The two largest groups of interneurons derived from the CGE are the bipolar CR and VIP subpopulations [13,53]. Recent data suggest that in addition to a CGE origin, there is a subpopulation of CR-positive interneurons that are derived from the dorsal MGE that co-express SST [58]. Moreover, immunocytochemical analysis of human fetal brain tissue at midgestation detected a population of neurons double-labeled for CR and NKX2.1 distributed within all layers of the cortex, further implying an MGE origin for a subset of CR-expressing interneurons [36,58]. The different origins of CR-positive interneurons may contribute to both the diversity of this population as well as additional sources of this subtype for the deep cortical layers of the developing neocortex.

In contrast to rodents, where PV-positive cells represent the majority of the interneuron population and CR-positive cells are much less abundant, several studies suggest that this ratio is reversed in humans as CR becomes the dominant interneuron subtype, accounting for at least 50% of all cortical GABAergic interneurons [33,59]. The significant increase in CR-expressing interneurons may be attributed to the proliferative expansion of the upper cortical layers of the neocortex, unique to primates and humans, that are populated by the later-born CR interneurons derived from the CGE and dorsal MGE [57]. Recently, establishing the origins of cortical interneurons in the human cortex has garnered much interest. The issue of whether the cortical ventricular (VZ) and subventricular (SVZ) zones are also a source of GABAergic inhibitory interneurons in addition to the ganglionic eminences remains controversial. By analyzing the expression patterns of progenitor cells using several key transcription factors associated with fate specification in the developing human and monkey telencephalon, Hansen et al. [34] and Ma et al. [35] concluded that the majority of interneuron progenitors are derived from the ganglionic eminences, with no evidence for a strong pallial contribution. In contrast, Radonjic et al. [36] observed *Shh*-expressing proliferative neural progenitor niches that are *NKX2.1*-positive in the cortical VZ and outer subventricular zone (oSVZ) in the cerebral cortex of monkeys and humans at midgestation. Additional reports propose a cortical origin for GABAergic neurons in the primate system, with the pallial progenitor pool primarily producing later born subpopulations, such as CR-expressing interneurons [60,61]. The inability to conduct definitive lineage tracing experiments within the human cortex makes it difficult to establish definitively whether these dorsally-derived progenitors are a source of cortical interneurons.

## 3. Shh Treatment for the Derivation of Interneurons from PSCs

Considerable advances in our understanding of the development, migration, and differentiation of cortical GABAergic interneurons in both rodent and primate brains have influenced in vitro studies aimed at generating cortical interneurons from PSCs. Numerous studies demonstrated neural induction from PSCs under serum-free conditions, producing cell types from both the dorsal and ventral forebrain [62,63,64,65]. Further manipulation of in vitro culture conditions of mouse ESCs showed that inhibition of Hedgehog signaling using cyclopamine resulted in a high percentage of excitatory pyramidal neurons at the expense of interneurons [62]. This provided the first clues that, as observed in vivo, Hedgehog signaling is important for interneuron specification in vitro. While loss of Shh activity results in regional cell fate conversion from MGE to CGE in vivo [14], inhibition of Shh signaling in PSC-derived cultures results in pallial cell specification and production of excitatory neurons [66]. This discrepancy could be because Shh knockouts in vivo remove function after the pallial versus subpallial fate is established, whereas loss of function approaches in PSC cultures act earlier, before this decision is made. Alternatively, pallial cell fate conversion in vitro could result from the use of proteins and/or small molecules, like the activin receptor-like kinase inhibitor SB431542 used in the dual SMAD neural differentiation protocol, that inhibit CGE differentiation [67]. The addition of exogenous Shh to differentiating cultures was sufficient to decrease expression of genes involved in specifying dorsal forebrain cell fates, while increasing the expression of ventral forebrain inhibitory interneuron progenitor genes like *NKX2.1* [63,65]. Using homologous recombination, Goulburn and colleagues generated an NKX2.1:GFP human ESC reporter cell line, allowing for live readout of cells committed to the MGE-like interneuron progenitor lineage [18].

The establishment of Shh as an important morphogen in the derivation of interneurons from PSCs has led many research groups to investigate the concentrations and duration of Shh treatment required for the specification of distinct interneuron subpopulations [17,19,20,21,22,64,66]. Increasing the concentration of Shh between 0 and 1000 ng/mL in vitro resulted in a significant increase in the percentage of *NKX2.1*-positive neural progenitors that subsequently went on to express the inhibitory neurotransmitter gamma-aminobutyric acid (GABA) [17,29]. In addition to Shh treatment, supplementation with Wnt inhibitors significantly increased the population of forebrain specific MGE-like *NKX2.1*-positive progenitors [19,20,21]. Varying the window and duration of Shh treatment from differentiation day 2–18 to differentiation day 10–18 boosted the proportion of *NKX2.1*-positive cells co-expressing the forebrain marker *FOXG1* to over 90% [20]. Notably, cells treated from day 10 to 18 of differentiation had an observable increase in the population of ventral forebrain *NKX2.1*-positive cells co-expressing *FOXG1* and *OLIG2*, characteristic of MGE-like interneurons, when compared to cells treated from days 6–18 [20], highlighting the importance of timing and duration of Shh treatment in regional specification of interneuron progenitors. Treatment with the Smoothened (Smo) agonist purmorphamine (Pur) from differentiation day 0 to 35 induced 90% NKX2.1:GFP expression [21]. Addition of the Smo agonist SAG between differentiation day 0 and day 25 produced approximately 28% *NKX2.1*-positive neural progenitors [19]. Gene expression profiling of *NKX2.1*-positive neural progenitors following treatment with Shh and/or Smo agonist treatment yielded enriched expression of forebrain specific, neuronal, GABAergic and MGE-like genes with very little dorsal telencephalic, LGE, CGE, dopaminergic or glutamatergic marker expression [20,21]. Mouse PSCs can also be specified to MGE derivatives using Shh treatment. Supplementation of differentiating mouse ESCs with 10nM Shh biased cultures toward subpallial cell fates as seen through expression of *GAD67*, *Dlx2*, and *Gsh2* [66]. Despite subpallial cell specification, the presence of significant *NKX2.1*-positive progenitors was only achieved following treatment with a higher concentration of 30nM Shh from days 3 to 9 of differentiation [66]. By changing the concentration, exposure time and length of culture in the presence of Shh, Tyson and colleagues generated enriched populations of SST or PV expressing interneurons [22]. Prior to transplantation into the mouse brain, PV biased neural cells required up to 17 days of culture in the absence of exogenous Shh, whereas SST biased cultures required a shorter culture duration of 12 days in the presence of exogenous Shh [22]. Ventralized progenitors expressing forebrain GABAergic interneuron markers can also be generated from murine and human fibroblasts without the use of Shh peptides or Smo agonists using a direct transcription factor reprogramming approach [68,69,70]. This outcome is likely due to the use of transcription factors acting downstream of *NKX2.1*, thus bypassing the need for Shh addition.

**Figure 2:**
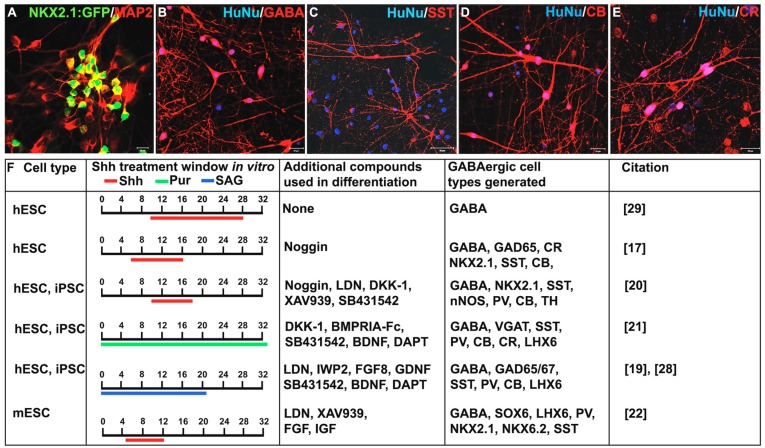
Comparison of protocols used to generate gamma-aminobutyric acid-containing GABAergic inhibitory interneurons from a PSC source. (**A**–**E**) *NKX2.1*-positive progenitors give rise to various interneuron subtypes in a co-culture system with mouse cortical astrocytes. Significant NKX2.1:GFP, MAP2, and calretinin (CR) protein expression observed after 10 weeks of co-culture and robust calbindin (CB), neuropeptide somatostatin (SST), and gamma-aminobutyric acid (GABA) expression seen after 17 weeks in vitro; (**F**) comparison of published protocols for the in vitro generation of GABAergic interneurons showing the time window of Sonic hedgehog (Shh) and Smoothened (Smo) agonist treatment, as well as additional exogenous compounds used in promoting neural differentiation. Scale bars: A = 10 μM, B–E = 20 μM. hESC = human embryonic stem cells, iPSC = induced pluripotent stem cells, mESC = mouse embryonic stem cells, Pur = purmorphamine.

Despite the relative ease with which GABAergic progenitors can be produced from PSCs, efficiently promoting their maturation into interneuron subtypes remains a challenge, particularly for human PSCs. Human PSC-derived *NKX2.1*-positive neural progenitors take an extended time, up to several months, to mature (reviewed in Tyson and Anderson, [71]). Co-culture in vitro with mouse cortical cells or astrocytes can promote differentiation into a variety of interneuron subtypes expressing GAD65/67, GABA (Figure 2A,B), SST (Figure 2C), CB (Figure 2D), CR (Figure 2E), PV, Reelin, VIP, or nNOS [17,19,20,21,28,54].

While the presence of numerous subtypes has been detected in vitro, the percentages of cells expressing specific neurochemical profiles varies greatly amongst the different protocols, with GABA expression ranging between 65% and 90%, SST from 2% to 50%, PV from <1% to 40%, CB from 25% to 65%, CR from 2% to 90%, and NPY from 0% to 10%. Discrepancies in the percentage of interneuron subtypes reported may be due to variation in the specific conditions and cell lines used by different groups (Figure 2B). Despite variability in the profile of subtypes produced, mature interneurons derived from these approaches are capable of making both excitatory and inhibitory connections in vitro [20]. Two months following transplantation of FACS isolated human PSC-derived GABAergic neural progenitors into the mouse brain, approximately 80% of the cells continue to express the migratory neuroblast marker doublecortin (DCX) [21]. These progenitors mature into SST, CR, PV and CB positive interneurons two to six months after transplantation [21,28,30]. Additionally, mouse ESC-derived interneurons displayed mature electrophysiological properties and morphologies similar to that of endogenous GABAergic interneurons [30]. Others demonstrated spontaneous postsynaptic currents (sPSC) [19,21] and the ability to induce or inhibit neuronal activity using optogenetic stimulation of GABAergic interneurons both in vitro and post transplantation [21,28].

## 4. Challenges to Future Clinical Applications Using PSC-Derived Interneurons

### 4.1. Human PSC-Derived Interneurons Require an Extended Maturation Timeline

In contrast to mouse PSC-derived interneuron progenitors, human-derived PSCs take an extended time to mature into functional interneurons, both in vitro and following transplantation. (Figure 1A,B, reviewed in Tyson and Anderson, [71]). Studies using disease-specific induced pluripotent stem cells (iPSCs) to model interneuron-related disorders and test therapeutic interventions in culture are dependent upon the in vitro protocols described (reviewed in Tyson and Anderson, [8]). The protracted time required for differentiation makes these studies challenging and costly. Therefore, approaches promoting accelerated maturation of interneurons would be extremely valuable in advancing the field. Cerebral organoid cultures may prove useful in developing appropriate strategies [24]. The several months required to observe mature interneuron phenotypes following transplantation into the rodent brain represents a concern for the development of interneuron-based transplantation therapies. Patients receiving transplants of PSC-derived interneuron progenitors will likely need to wait several months before experiencing any potential positive effects. To speed up the time required for therapeutic efficacy, grafting of more mature cells or addition of factors that promote terminal differentiation post-transplantation may be required.

### 4.2. Selective and Efficient Generation of Interneuron Subtypes

PSC-derived interneuron progenitors differentiate into a variety of interneuron subtypes in vitro or following transplantation into the rodent brain. For human PSCs in particular, it has proven difficult to direct production of specific interneuron subtypes. Interneuron-based disorders can be characterized by the loss of particular interneuron subtype(s), and therefore protocols that are subtype-specific would most accurately provide in vitro disease models or produce desired cell-based therapies. For example, selective loss or damage to PV and SST interneurons characterizes temporal lobe epilepsy. Production of abundant PV-expressing interneurons from human PSCs has been challenging, perhaps due to the observation that their maturation is activity dependent [72,73,74]. Consequently, GABAergic progenitors might require co-culture with excitatory cell types to successfully induce PV expression. Tyson et al. investigated the production of PV- versus SST-expressing interneurons from mouse ESCs and observed that endogenous levels of Shh in differentiating cultures is sufficient to generate primarily PV expressing interneurons, while the addition of exogenous Shh biased cultures towards SST expression [22]. It remains to be seen whether conditions for low and high Shh signaling support the balance between PV and SST interneurons from human PSCs. Apart from deriving PV and SST interneuron subtypes, there is considerable interest in generating CR cortical interneurons from human PSCs based upon the high percentage of CR-expressing cells in the human cortex, and their vulnerability in diseased states [75]. The serotonin receptor 5-HT3R has been identified as a marker for CGE-derived interneurons in rodents, and may prove useful in identifying PSC-derived CR interneurons [76].

### 4.3. Genetic Modification of Cell Lines Used for Clinical Applications

Enrichment of interneuron progenitors for long-term differentiation and transplantation studies has largely been achieved using fluorescence activated cell sorting (FACS) of genetically modified cell lines expressing fluorescent reporters [17,20,21]. However, the use of genetically modified lines poses a major barrier for use in clinical applications. An alternative FACS-based approach is the use of antibodies against cell surface antigens specific for MGE-like interneuron progenitors. To date, there are no known cell surface antigens that characterize MGE-like GABAergic neural progenitors. For example, antibodies directed against polysialylated-neural cell adhesion molecule (PSA-NCAM), a cell surface marker present on mature migratory neural cells, have been utilized to isolate neural progenitors for transplantation [19,28]. Enrichment of NKX2.1:GFP and PSA-NCAM double positive cells allowed for selection of more mature cells for transplantation, which greatly reduced the formation of neural tumors [21]. Identification of an MGE-specific cell surface molecule would facilitate designing a useful interneuron-selection protocol.

## 5. Conclusions

Protocols for generating cortical interneuron progenitors from PSCs are now well established and involve the measured use of Shh agonists. Future goals include fine-tuning and extending these approaches to promote selective production of mature interneuron subtypes in vitro. These advances would further our understanding of interneuron-based diseases and potential therapeutic approaches by providing PSC-based assays for interneuron differentiation, maturation, and function that could also be used as a drug screening platform. PSC-derived interneurons are also being used for cell-based therapies designed to replenish supplies of GABAergic cells in animal models of interneuron disorders.

## Abbreviations

The following abbreviations are used in this manuscript:

CNS	Central nervous system
SST	Somatostatin
PV	Parvalbumin
CR	Calretinin
CB	Calbindin
NPY	Neuropeptide-Y
VIP	Vasoactive intestinal peptide
GABA	Gamma-Aminobutyric acid
GABAergic	Gamma-aminobutyric acid-containing interneurons
GAD	Glutamic acid decarboxylase
nNOS	Neuronal nitric oxide synthase
ASD	Autism spectrum disorders
AD	Alzheimer’s disease
MGE	Medial ganglionic eminence
CGE	Caudal ganglionic eminence
LGE	Lateral ganglionic eminence
PSCs	Pluripotent stem cells
Shh	Sonic hedgehog
Smo	Smoothened
SAG	Smoothened agonist
VZ	Ventricular zone
SVZ	Subventricular zone
oSVZ	Outer subventricular zone
POA	Preoptic area
FACS	Fluorescence activated cell sorted
iPS	Induced pluripotent stem
iPSC	Induced pluripotent stem cell
ESC	Embryonic stem cell
sPSC	spontaneous postsynaptic currents
DCX	Doublecortin
PSA-NCAM	Polysialylated-neural cell adhesion molecule
